# Supplementary material for: Paclitaxel compromises nuclear integrity in interphase through SUN2-mediated cytoskeletal coupling
Source: J Cell Sci. 2026 Jan 20;139(12):jcs264494. doi: 10.1242/jcs.264494 (PMC12863304; doi:10.1242/jcs.264494)
Supplement: Supplementary information [file joces-139-264494-s1.pdf]

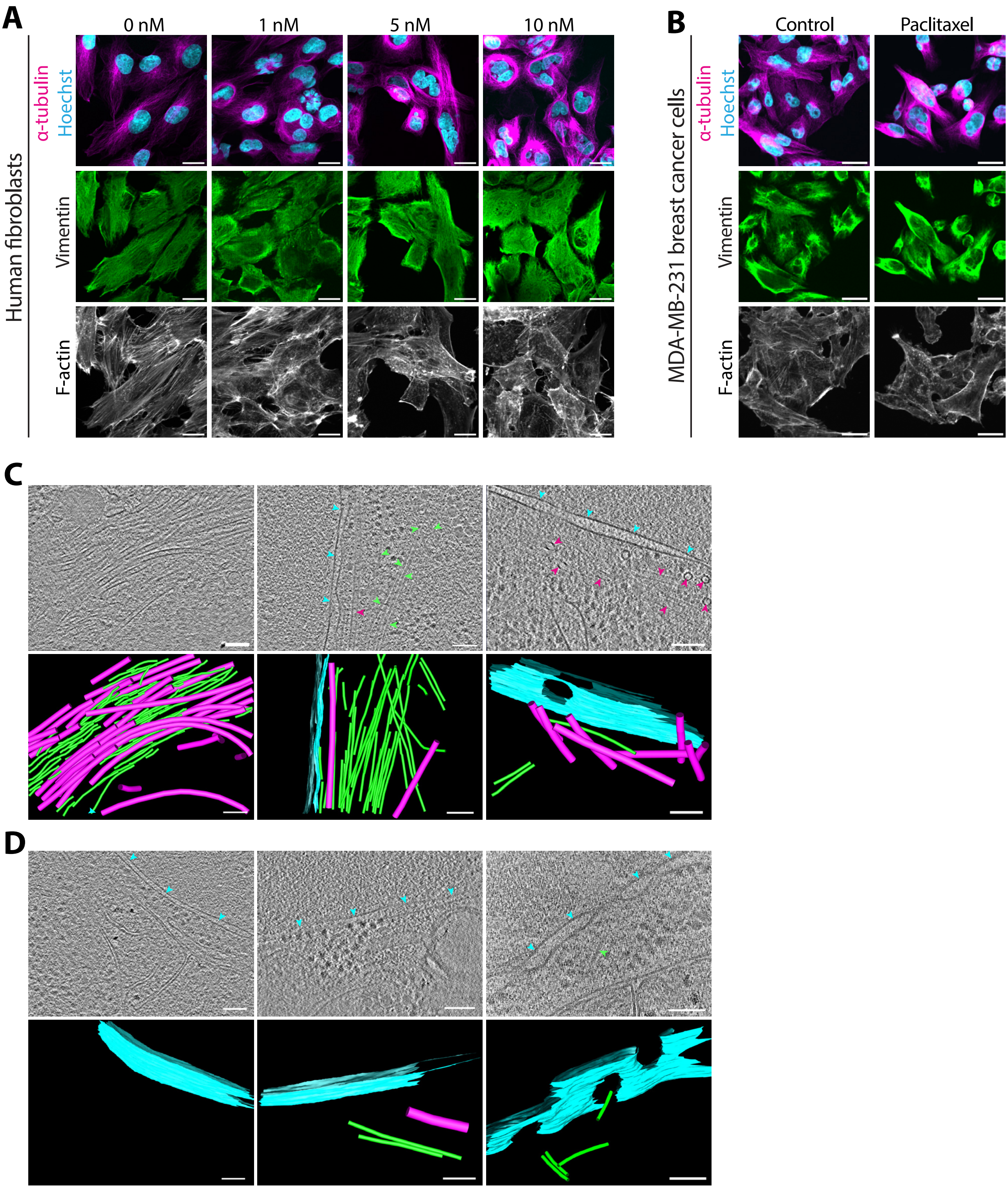

Fig. S1. Paclitaxel-induced cytoskeletal reorganisation around the nucleus in interphase.
(A) Confocal micrographs of human fibroblasts fixed after 16 h incubation in 0, 1, 5, or 10 nM paclitaxel. DNA was stained using Hoechst (cyan), microtubules using α-tubulin immunofluorescence (magenta), vimentin using immunofluorescence (green), and F-actin using phalloidin (white). Scale bars = 20 μm. **(B)** Confocal micrographs of breast cancer MDA-MB-231 cells fixed after 16 h incubation in control media or 5 nM paclitaxel. DNA was stained using Hoechst (cyan), microtubules using α-tubulin immunofluorescence (magenta), vimentin using immunofluorescence (green), and F-actin using phalloidin (white). Scale bars = 20 μm. **(C)** Top panels show 2D slices of reconstructed tomograms from paclitaxel-treated human fibroblasts. Bottom panels show segmentations from these tomograms of microtubules (magenta), vimentin filaments (green), and the NE (cyan), which are marked with arrowheads of the same colour in the tomogram slice. Scale bars = 100 nm. **(D)** As in (C), but with control cells.

Journal of Cell Science • Supplementary information

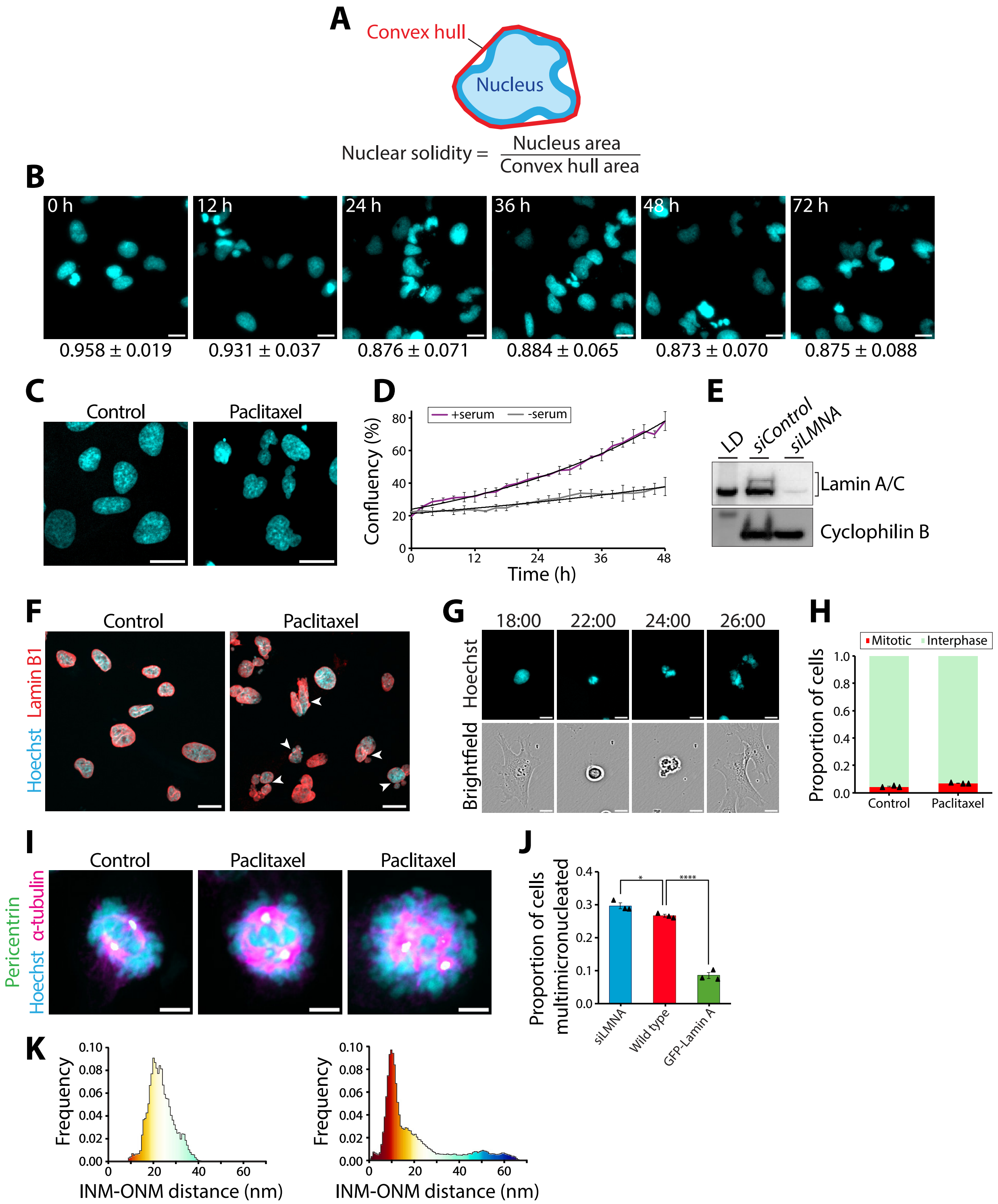

Fig. S2. Paclitaxel results in nuclear deformation.
(A) Nuclear solidity is used to quantify nuclear deformation and is defined as the nucleus area (blue) divided by the convex hull area of the nucleus (red) (Janssen et al. 2022). **(B)** Example of images used for nuclear solidity quantification in Fig. 2A. Frames from a live-cell movie of Hoechst-stained nuclei 0-72 h after addition of 5 nM paclitaxel are shown, together with the mean nuclear solidity measurements ± the standard deviation from these images. **(C)** Representative images of serum-starved cells following 16 h incubation in control media or 5 nM paclitaxel, that were used for the quantification in Fig. 2C. **(D)** Cell confluency of cells cultured over 48 h in complete medium (+serum), or serum-starved medium (-serum). Using non-linear regression to fit exponential growth curves (black) showed that the doubling time was significantly increased from 28.7 h in complete medium to 58.9 h in serum-starved medium. **(E)** Western blot confirming knockdown of Lamin A and Lamin C following transfection with siRNA against LMNA (siLMNA), compared to with control siRNA (siControl). Cyclophilin B was used as the loading control. Lane 1 shows protein ladder (LD; 62 kDa for Lamin A/C panel, and 28 kDa for Cyclophilin B panel). **(F)** Confocal micrographs of cells fixed after 24 h incubation in control media or 5 nM paclitaxel, with the nucleus stained using Hoechst (cyan) and Lamin B1 immunofluorescence (red). Multimicronucleated cells are marked with arrowheads. Scale bars = 20 µm. **(G)** Live cell imaging showing a Hoechst-stained cell undergoing mitosis in the presence of 5 nM paclitaxel 18-26 h after paclitaxel addition. Scale bars = 20 µm. **(H)** Bar graph showing the proportion of cells that were in interphase (green) versus mitosis (red) following 30 h incubation in 5 nM paclitaxel or control media. Mitotic cells were identified by the presence of condensed chromosomes and a mitotic spindle

Journal of Cell Science • Supplementary information

following staining for DNA using Hoechst and microtubules using α-tubulin immunofluorescence. Error bars represent the s.e.m. from three biological repeats (n=3) which are marked with triangles. **(I)** Confocal micrographs of cells fixed after 24 h incubation in control media or 5 nM paclitaxel. Cells were stained for DNA using Hoechst (cyan), microtubules using α-tubulin immunofluorescence (magenta), and centrosomes using pericentrin immunofluorescence (green). Scale bars = 5 µm. **(J)** Bar graph comparing the proportion of wild-type (red), Lamin A/C knockdown (siLMNA – blue), and GFP-Lamin A overexpressing (green) cells that were multimicronucleated following 24 h incubation in 5 nM paclitaxel. Multimicronucleated cells were identified from confocal micrographs of cells stained for DNA using Hoechst. Error bars represent the s.e.m. from three biological repeats (n=3) which are marked with triangles, each with at least 60 cells. Statistical analysis: t-test versus wild type. siLMNA: P=0.0346 (*); GFP-Lamin A: P=4.86 x 10-5 (****). **(K)** Histograms showing the ONM-INM distance from the control tomogram (left) and the paclitaxel tomogram (right) in Fig. 2G.

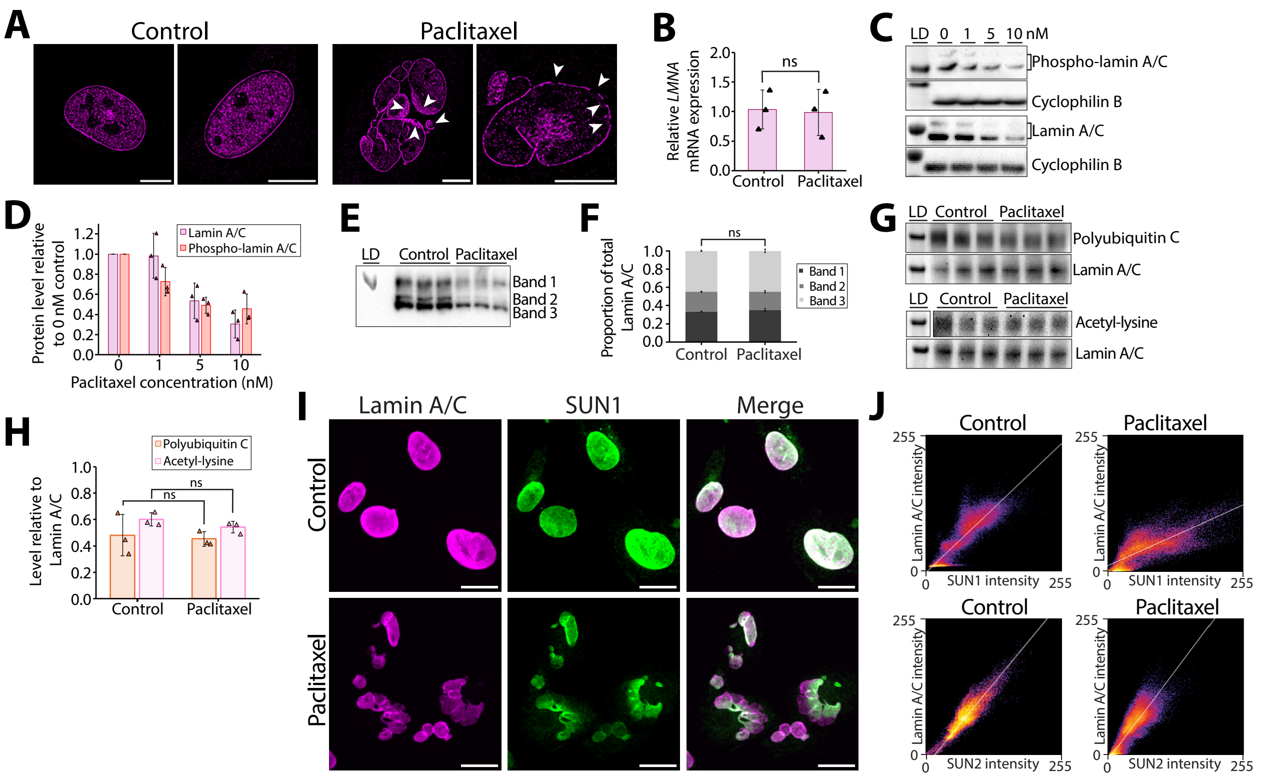

Fig. S3. Paclitaxel treatment results in aberrant organisation and decreased levels of Lamin A/C and SUN2. (A) STORM images of cells fixed after 16 h incubation in control media or 5 nM paclitaxel. Lamin A/C was stained using immunofluorescence (magenta). Scale bars = 10 µm. Holes in the nuclear lamina are marked with arrowheads. **(B)** Bar chart showing the relative mRNA expression levels of LMNA between control cells and cells treated with 5 nM paclitaxel for 24 h, calculated using the 2−ΔΔCt method with GAPDH as the housekeeping gene control. Error bars show the standard deviation from three biological repeats (n=3) which are marked with triangles, each with three technical repeats. Statistical test: t-test, P=0.8742. **(C)** Western blots for phospho-Ser404 Lamin A/C and Lamin A/C (from Fig. 3B) of whole cell lysates following 16 h incubation in media containing 0, 1, 5, or 10 nM paclitaxel. Cyclophilin B was used as a loading control. Lane 1 shows protein ladder (LD; 62 kDa for phospho-lamin A/C panel, 28 kDa for Cyclophilin B upper panel, 75 kDa for Lamin A/C panel, and 25 kDa for Cyclophilin B lower panel). **(D)** Quantification of Lamin A/C and phospho-Lamin A/C levels from (C). Each band was normalised to the corresponding Cyclophilin B loading control. Error bars represent the standard deviation from three biological repeats (n=3) which are marked with triangles. **(E)** Western blot for Lamin A/C of whole cell lysates following 16 h incubation in control media or 5 nM paclitaxel. The lysates were run on a Phos-tag gel to separate phosphorylated and non-phosphorylated Lamin A/C. Three biological repeats were used for each condition. Lane 1 shows protein ladder (LD; 62 kDa). **(F)** Quantification of the proportion of total Lamin A/C protein in each of the three bands from (E). Errors bars represent the standard deviation from the three biological repeats (n=3). Statistical test: proportions were first transformed using a centered log-ratio transformation, followed by a MANOVA with Pillai's trace test, resulting in no significant multivariate effect (P=0.5499). **(G)** Western blot for Polyubiquitin C and acetyl-lysine following pull-down of Lamin A/C from whole cell lysates of control cells or cells treated with 5 nM paclitaxel for 16 h. Three biological repeats were used for each condition. To control for Lamin A/C protein levels, the same membranes were stripped and blotted for Lamin A/C. **(H)** Quantification of Polyubiquitin C and acetyl-lysine from (G), with each band normalised to Lamin A/C. Error bars represent the standard deviation from three biological repeats (n=3) which are marked with triangles. Statistical analysis: t-test control versus paclitaxel: Polyubiquitin C P=0.7840; acetyl-lysine P=0.1988. **(I)** Confocal micrographs of cells fixed after 16 h incubation in control media or 5 nM paclitaxel. Cells were stained for Lamin A/C (magenta) and SUN1 (green) using immunofluorescence. Scale bars = 20 µm. **(J)** Co-localisation between Lamin A/C and SUN1/SUN2 visualised using scatterplots of Lamin A/C fluorescence intensity versus SUN1/SUN2 fluorescence intensity for each pixel of images in (I) (SUN1) and Fig. 3D (SUN2).

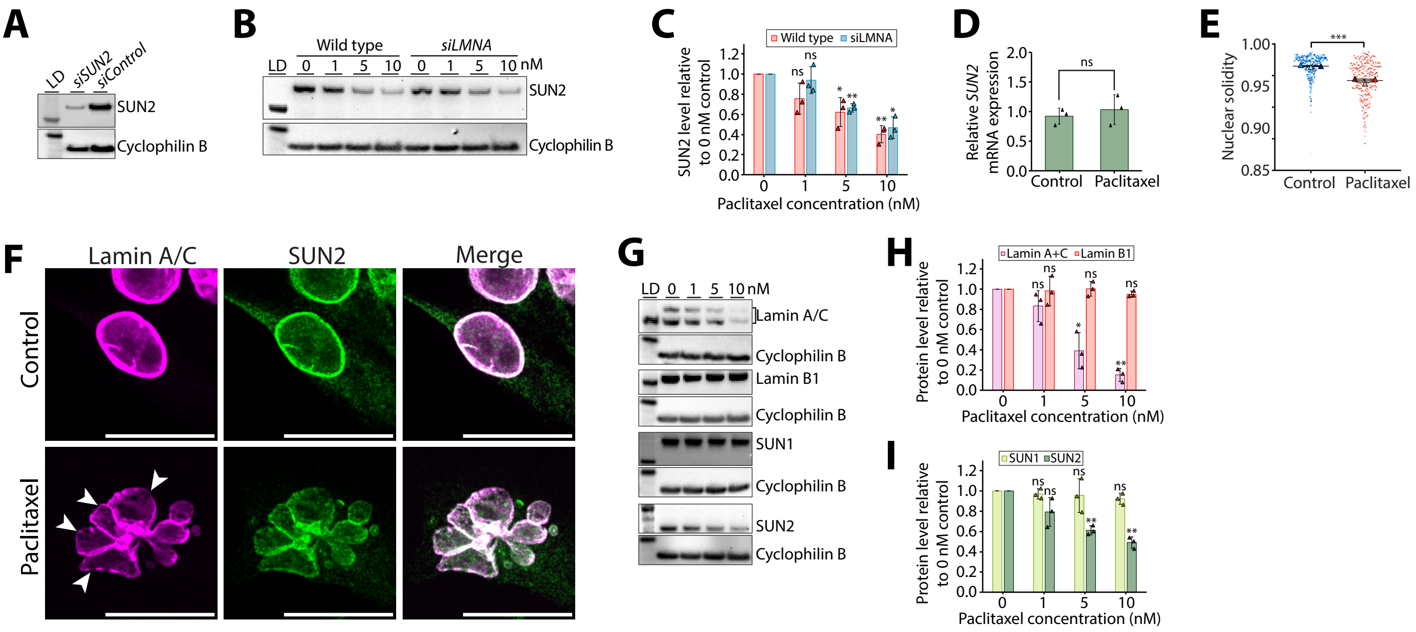

Fig. S4. The paclitaxel-induced decrease in Lamin A/C levels occurs via SUN2.
(A) Western blot confirming knockdown of SUN2 following transfection with siRNA against SUN2 (siSUN2), compared to control siRNA (siControl). Cyclophilin B was used as the loading control. Lane 1 shows protein ladder (LD; 98 kDa for SUN2 panel, and 28 kDa for Cyclophilin B panel). **(B)** Western blots for SUN2 of whole cell lysates from wild-type or Lamin A/C knockdown (siLMNA) cells following 16 h incubation in media containing 0, 1, 5, or 10 nM paclitaxel. Cyclophilin B was used as a loading control. Lane 1 shows protein ladder (LD; 62 kDa for SUN2 panel, and 28 kDa for Cyclophilin B panel). **(C)** Quantification of SUN2 levels from (B). Each band was normalised to the corresponding Cyclophilin B loading control. Error bars represent the standard deviation from three biological repeats (n=3) which are marked with triangles. Statistical analysis: one-sample t-test with null hypothesis mean = 1. Wild type: 1 nM P=0.1112; 5 nM P=0.0445; 10 nM P=0.0070. siLMNA: 1 nM P=0.5221; 5 nM P=0.0053; 10 nM P=0.0132. **(D)** Bar chart showing the relative mRNA expression levels of SUN2 between control cells and cells treated with 5 nM paclitaxel for 24 h, calculated using the 2−ΔΔCt method with GAPDH as the housekeeping gene control. Error bars show the standard deviation from three biological repeats (n=3) which are marked with triangles, each with three technical repeats. Statistical test: t-test, P=0.5264. **(E)** Quantification of nuclear solidity of breast cancer MDA-MB-231 cells in control media, or after 16 h incubation in 5nM paclitaxel. Error bars represent the s.e.m. from three biological repeats (n=3), each with more than 100 cells. The datapoint from each cell is marked with a dot colour-coded according to the biological replicate it came from, with the mean of each biological repeat marked with a triangle of the same colour. Statistical test: t-test, P=0.0008. **(F)** Confocal micrographs of breast cancer MDA-MB-231 cells fixed after 16 h incubation in control media or 5 nM paclitaxel. Cells were stained for Lamin A/C (magenta) and SUN2 (green) using immunofluorescence. Scale bars = 20 µm. **(G)** Western blots for Lamin A/C, Lamin B1, SUN1, and SUN2 of whole cell lysates from breast cancer MDA-MB-231 cells following 16 h incubation in media containing 0, 1, 5, or 10 nM paclitaxel. Cyclophilin B was used as a loading control. Lane 1 shows protein ladder (LD; 62 kDa for Lamin A/C and Lamin B1 panels, 98 kDa for SUN1 and SUN2 panels, and 28 kDa for Cyclophilin B panels). **(H)** Quantification of Lamin A/C and Lamin B1 protein levels from (G). Each band was normalised to the corresponding Cyclophilin B loading control. Error bars represent the standard deviation from three biological repeats (n=3) which are marked with triangles. Statistical analysis: one-sample t-test with null hypothesis mean = 1. Lamin A/C: 1 nM P=0.1981; 5 nM P=0.0279; 10 nM P=0.0019. Lamin B1: 1 nM P=0.8559; 5 nM P=0.9317; 10 nM P=0.1025. **(I)** Quantification of SUN1 and SUN2 protein levels from (G). Each band was normalised to the corresponding Cyclophilin B loading control. Error bars represent the standard deviation from three biological repeats (n=3) which are marked with triangles. Statistical analysis: one-sample t-test with null hypothesis mean = 1. SUN1: 1 nM P=0.4215; 5 nM P=0.6866; 10 nM P=0.1322. SUN2: 1 nM P=0.1263; 5 nM P=0.0043; 10 nM P=0.0041. Ns = non-significant = P>0.05; * P<0.05; ** P<0.01, *** P<0.001.

Journal of Cell Science • Supplementary information

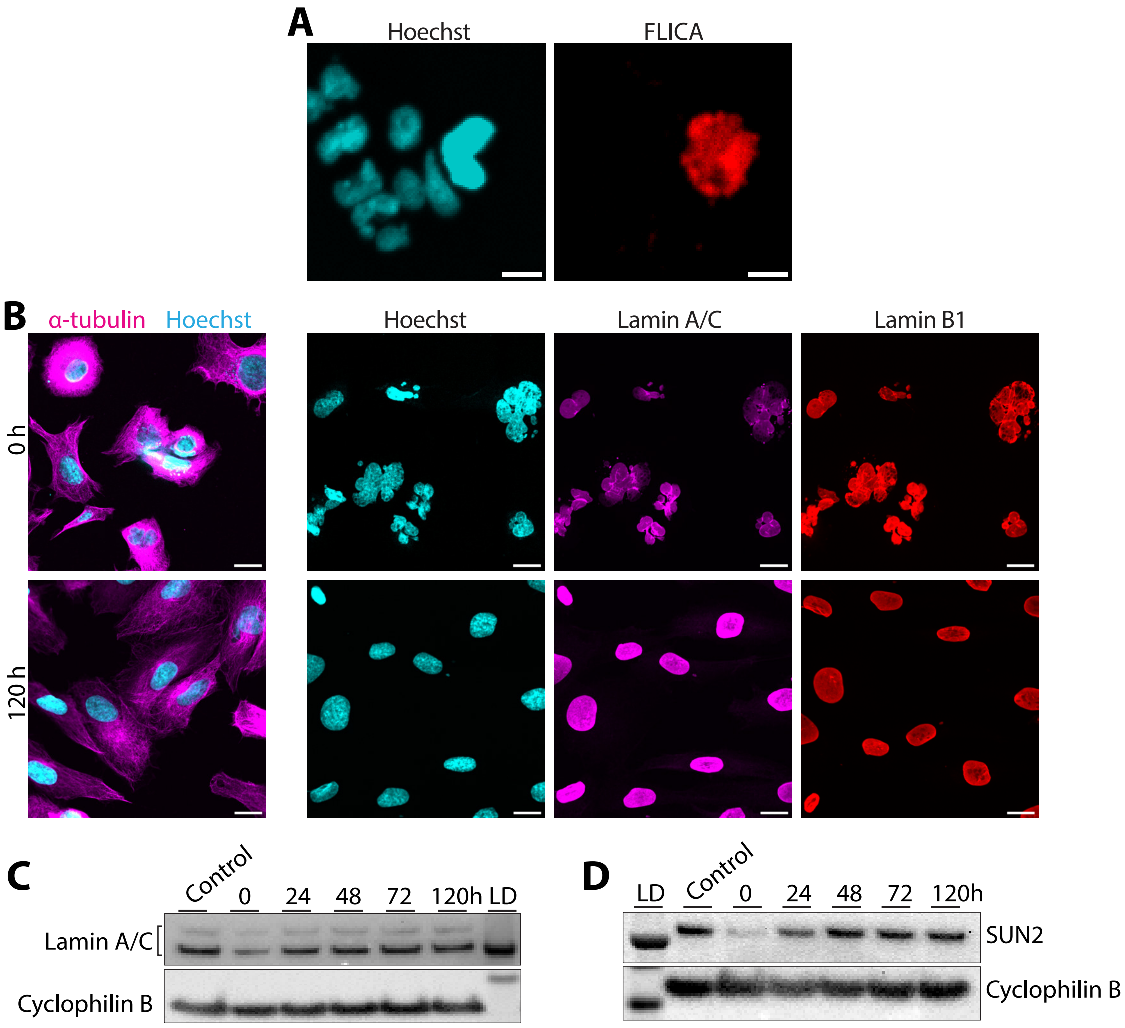

Fig. S5. Lamin A/C expression level affects cell sensitivity to and recovery from paclitaxel.
(A) Example micrograph used in the cell viability analysis in Fig. 6B. Cells were stained for DNA using Hoechst (cyan), and active caspases using FLICA (red). Scale bars = 10 µm. **(B)** Confocal micrographs of cells fixed 0 or 120 h after removal of 5 nM paclitaxel, showing that the microtubule organisation and nuclear lamina recover following paclitaxel removal. Cells were stained for DNA using Hoechst (cyan), and microtubules using α-tubulin immunofluorescence (magenta); or for DNA using Hoechst (cyan), and Lamin A/C (magenta) and Lamin B1 (red) using immunofluorescence. Scale bars = 20 µm. **(C-D)** Western blots for Lamin A/C (C) and SUN2 (D) of whole cell lysates 0, 24, 48, 72, or 120 h following removal of 5 nM paclitaxel, showing that Lamin A/C and SUN2 protein levels recover following paclitaxel removal. Untreated cells were used as a control (Control). Cyclophilin B was used as a loading control. LD lane = protein ladder (62 kDa for Lamin A/C panel, 28 kDa for Cyclophilin B panel in (C), 75 kDa for SUN2 panel, and 20 kDa for Cyclophilin B panel in (D)).

Journal of Cell Science • Supplementary information

Fig. S2E

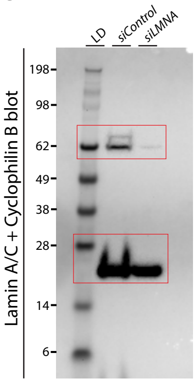

Fig. 3B,C and Fig. 3F,G and Fig. S3C,D

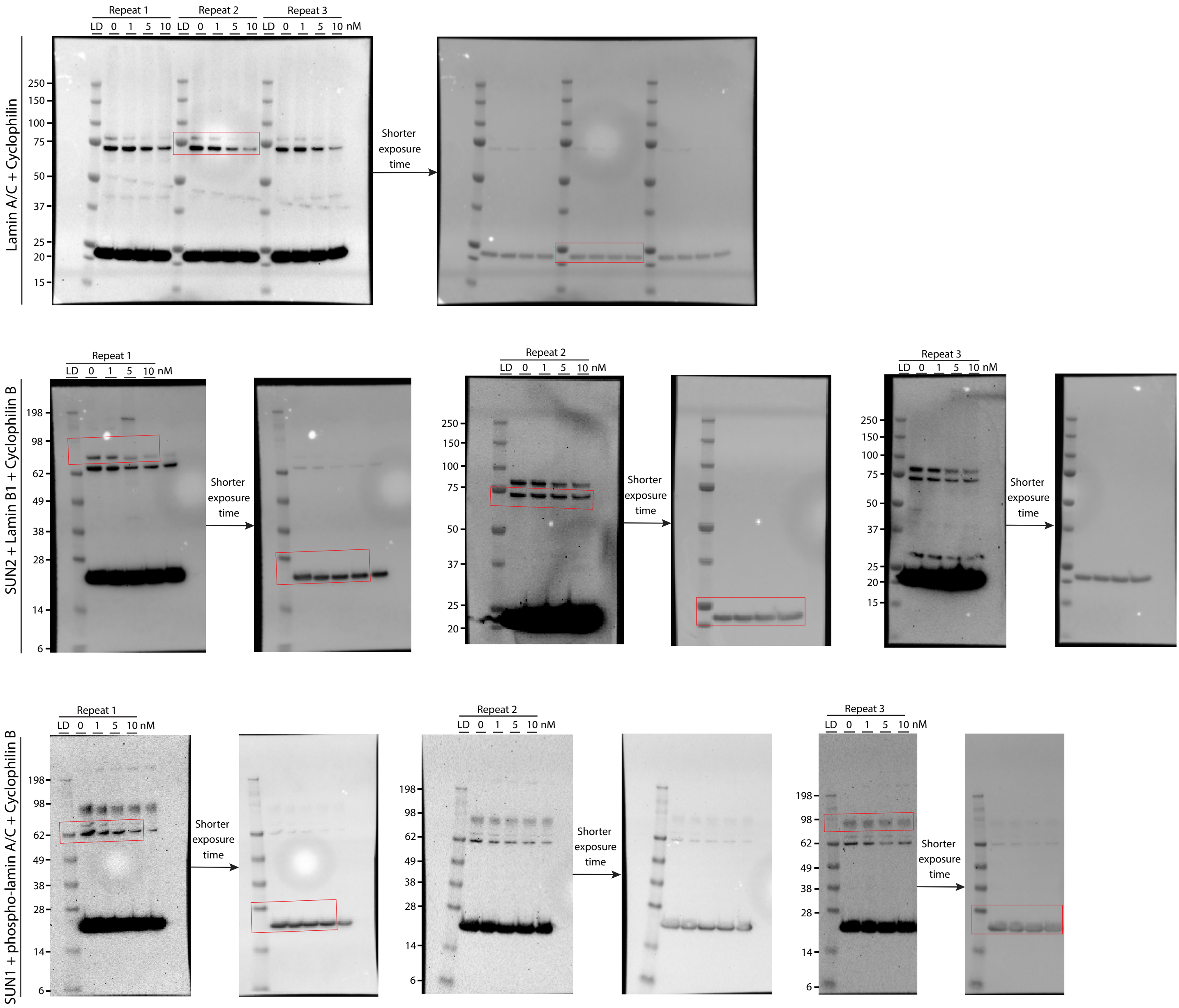

Journal of Cell Science • Supplementary information

Fig. 4A,B

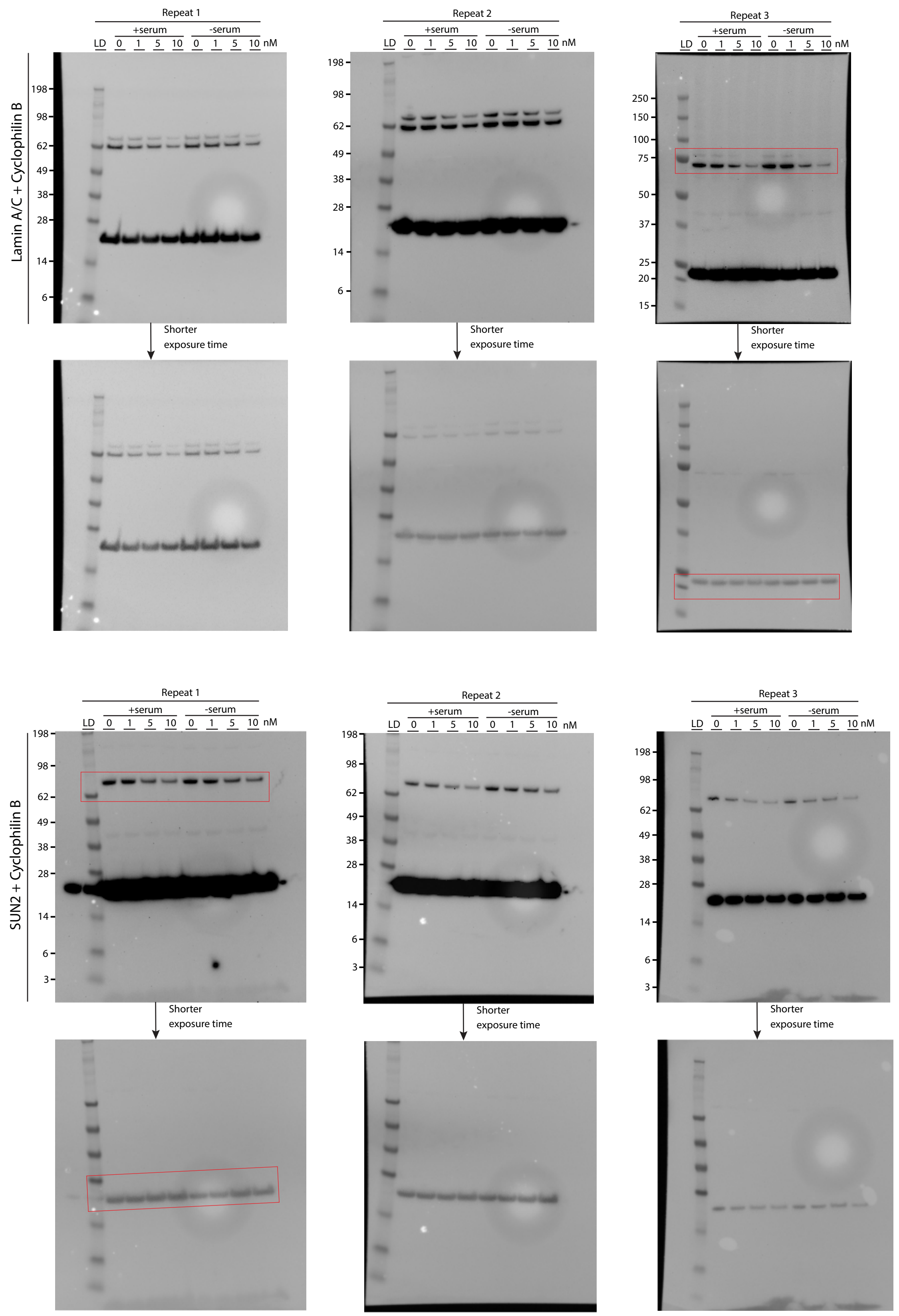

Journal of Cell Science • Supplementary information

Fig. 4D,E

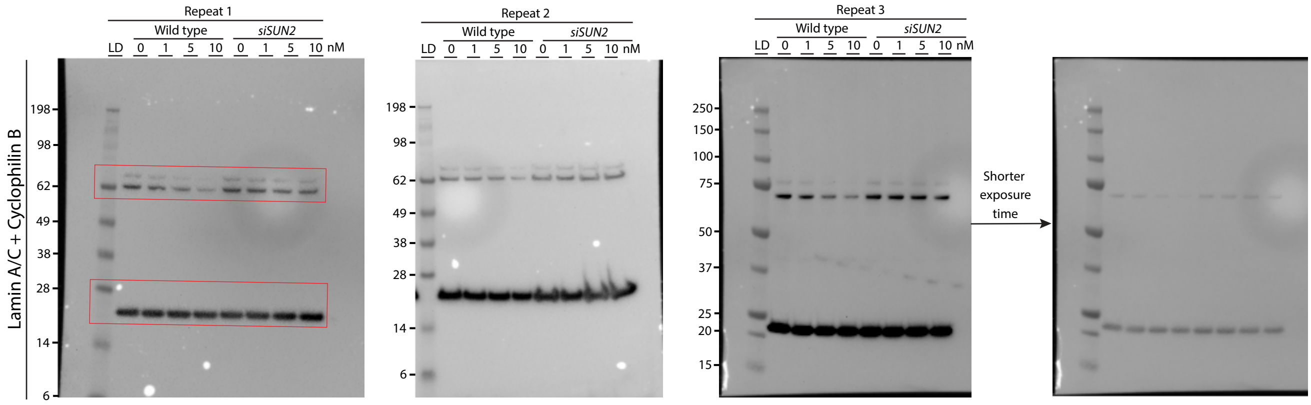

Fig. 4F,G

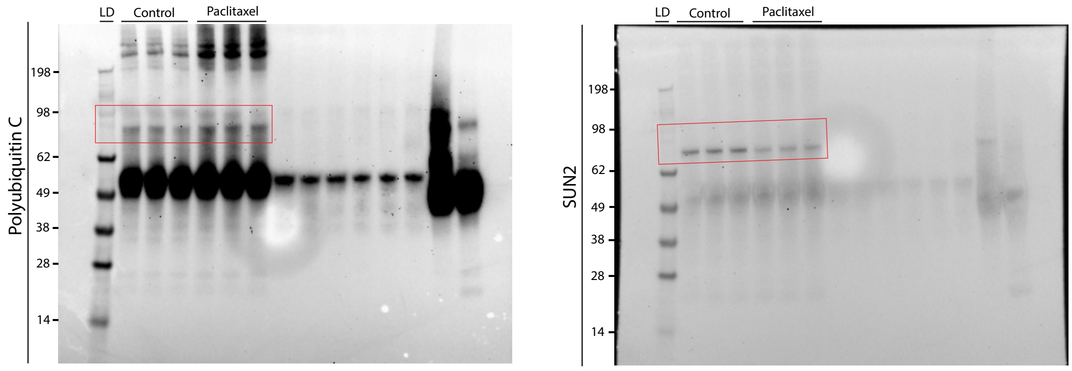

Fig. S3C,D

For phospho-lamin A/C and corresponding Cylophilin B loading control, see Fig. 3F,G blots.
The Lamin A/C and corresponding Cylophilin B loading control blots are as in Fig. 3B.

Fig. S3E,F

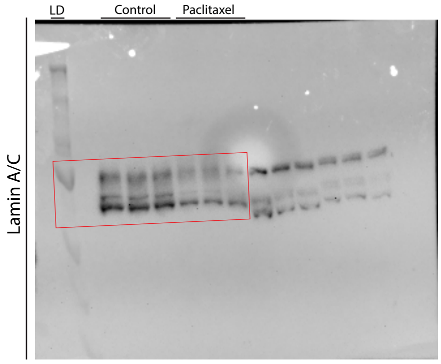

Fig. S3G,H

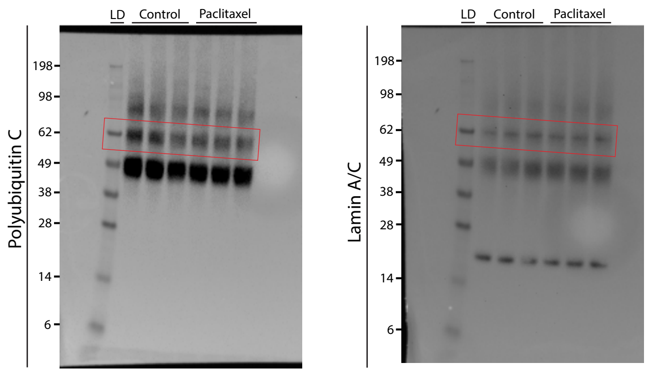

Journal of Cell Science • Supplementary information

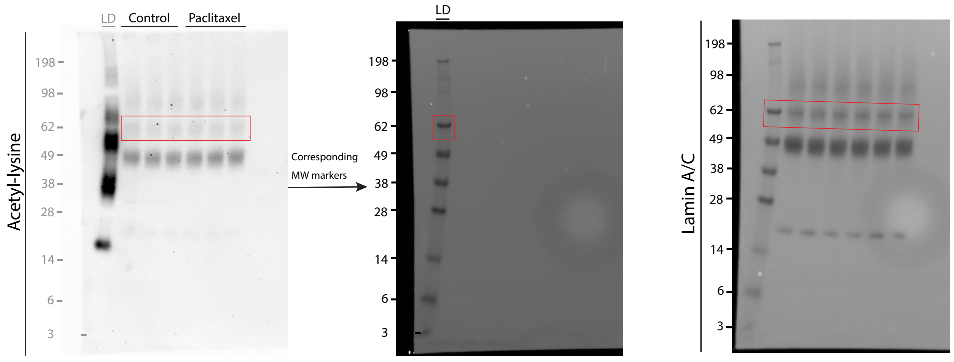

Fig. S4A

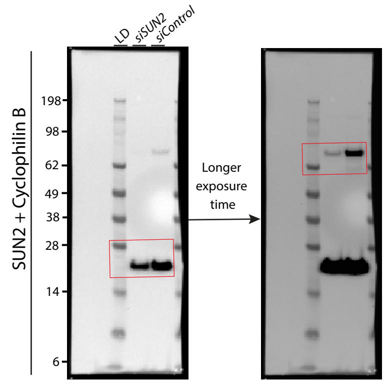

Fig. S4B,C

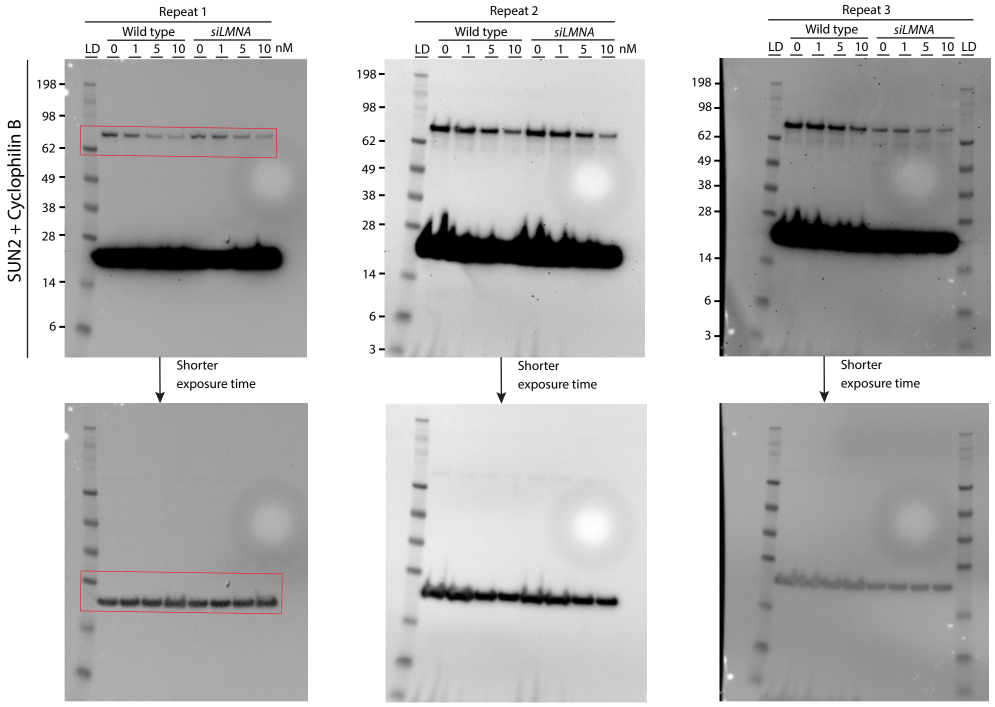

Journal of Cell Science • Supplementary information

Fig. S4G,H,I

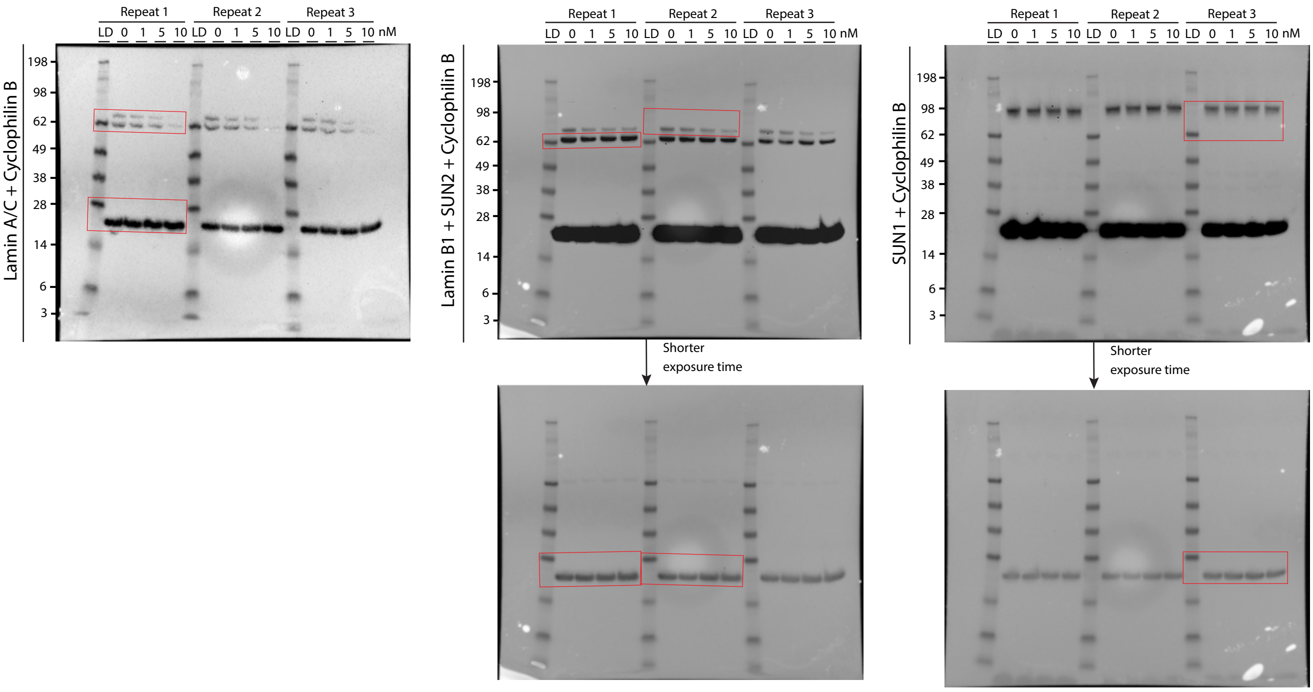

Fig. S5C

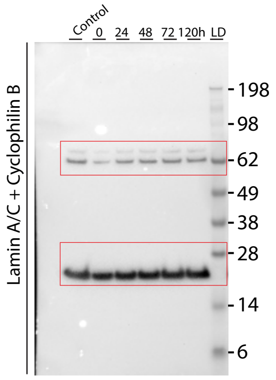

Fig. S5D

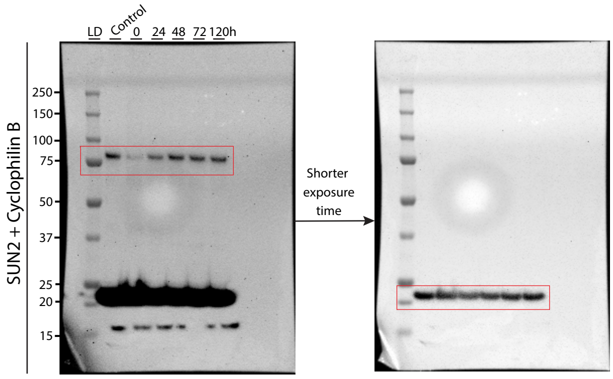

Fig. S6. Blot transparency

Uncropped Western blot scans from the indicated figure, including biological repeats used for quantification. The primary antibody targets are shown on the left. LD lane contains the protein ladder for which the molecular weights in kDa are annotated. The areas of the blots used for figures are marked with a red rectangle. Note that the camera channel used to image the molecular weight markers contains a circular defect, but this is not present in the chemiluminescence channel so does not affect quantification (see the Fig. S3G/H uncropped acetyl-lysine blot where the molecular weight channel and chemiluminescence channel are displayed separately).

Table S1. Details of siRNAs used for knockdowns

siRNA	Target mRNA	Source
siLMNA	*LMNA*	Ambion 4392420 Assay ID s530951
siSUN2	*SUN2*	Ambion 4392420 Assay ID s24467
siControl	N/A	Ambion 4390844

Table S2. Details of antibodies used for immunofluorescence, Western blotting, and pull-downs.

Antibody	Dilution (IF - immunofluorescence; WB - Western blot; IP - pull-down)	Source
Mouse anti-α tubulin	IF: 1:100	Invitrogen A11126
Rabbit anti-vimentin	IF: 1:200	Abcam ab92547
Rabbit anti-Lamin B1	IF: 1:200; WB: 1:2,000	Abcam ab16048
Mouse anti-Lamin A/C	IF: 1:200; WB: 1:1,000	Santa Cruz sc7292
Rabbit anti-Lamin A/C	WB: 1:1,000; IP: 1:50	Abcam ab108595
Mouse anti-phospho-Ser404 Lamin A/C	WB: 1:1,000	Sigma ABT1387
Rabbit anti-SUN1	IF: 1:200; WB: 1:2,000	Sigma HPA008346
Rabbit anti-SUN2	IF: 1:500; WB: 1:2,000; IP: 1:50	Abcam ab124916
Rabbit anti-Cyclophilin B	WB: 1:2,000	Abcam ab16045
Rabbit anti-Polyubiquitin C	WB: 1:500	Abcam ab104455
Mouse anti-acetyl lysine	WB: 1:500	Abcam ab22550
Donkey anti-Mouse IgG – Alexa 555	IF: 1:500	Abcam ab150110
Donkey anti-Mouse IgG – Alexa 647	IF: 1:500	Abcam ab150111
Donkey anti-Rabbit IgG – Alexa 555	IF: 1:500	Abcam ab150074
Donkey anti-Rabbit IgG – Alexa 647	IF: 1:500	Abcam ab181347
Goat anti-Rabbit IgG - HRP	WB: 1:15,000	Abcam ab6721
Goat anti-Mouse IgG - HRP	WB: 1:10,000	Abcam ab97023

Table S3. Primer sequences used for RT-qPCR

mRNA	Forward primer	Reverse primer
LMNA	AGAACATCTACAGTGAGGAG	CAGAATAAGTCTTCTCCAGC
SUN2	AGCCTTCAGATTCTCTTCAG	ATTTCCTCTTCAAGGTCCTG
GAPDH	TCGGAGTCAACGGATTTG	CAACAATATCCACTTTACCAGAG
